# Unraveling the adult cell progeny of early postnatal progenitor cells

**DOI:** 10.1038/s41598-020-75973-y

**Published:** 2020-11-04

**Authors:** Rebeca Sánchez-González, Nieves Salvador, Laura López-Mascaraque

**Affiliations:** Instituto Cajal-CSIC, 28002 Madrid, Spain

**Keywords:** Adult neurogenesis, Neural progenitors

## Abstract

NG2-glia, also referred to as oligodendrocyte precursor cells or polydendrocytes, represent a large pool of proliferative neural cells in the adult brain that lie outside of the two major adult neurogenic niches. Although their roles are not fully understood, we previously reported significant clonal expansion of adult NG2-cells from embryonic pallial progenitors using the StarTrack lineage-tracing tool. To define the contribution of early postnatal progenitors to the specific NG2-glia lineage, we used NG2-StarTrack. A temporal clonal analysis of single postnatal progenitor cells revealed the production of different glial cell types in distinct areas of the dorsal cortex but not neurons. Moreover, the dispersion and size of the different NG2 derived clonal cell clusters increased with age. Indeed, clonally-related NG2-glia were located throughout the corpus callosum and the deeper layers of the cortex. In summary, our data reveal that postnatally derived NG2-glia are proliferative cells that give rise to NG2-cells and astrocytes but not neurons. These progenitors undergo clonal cell expansion and dispersion throughout the adult dorsal cortex in a manner that was related to aging and cell identity, adding new information about the ontogeny of these cells. Thus, identification of clonally-related cells from specific progenitors is important to reveal the NG2-glia heterogeneity.

## Introduction

NG2-cells or NG2-glia, also known as oligodendrocyte precursor cells (OPCs), represent a rapidly responding reservoir of new oligodendrocytes^1–3^. These cells express both the neuron-glia antigen 2 (NG2) chondroitin sulfate proteoglycan and the alpha receptor for platelet-derived growth factor (PDGFRα), as well as the oligodendrocyte marker, Olig2 (oligodendrocyte transcription factor 2). NG2-glia have been attributed different names over the years. Here, the terms NG2-glia or NG2-cells are used to define the glial cells expressing NG2, Olig2 and PDGFRα, in order to distinguish them from non-glial cells that also express NG2^4,5^. In addition to oligodendrocytes, NG2-glia have the potential to generate a variety of cell types in vitro and in vivo, like astrocytes and neurons, both at postnatal or adult stages^4,6–8^. While the adult differentiation of these cells remains unclear^9–11^, several studies have focused on the capacity of NG2-glia to reprogramme into neurons in vivo^12,13^, opening the window to develop new therapeutic strategies involving this cell type.

NG2-glia come into close contact with other glial cells and neurons^14^, and they can receive direct synaptic inputs from neurons^15^, adjusting their behavior in response to neuronal activity. Furthermore, these cells are subject to the effects of a huge pool of transcription factors, neurotrophins, growth factors, metalloprotease inhibitors, cell adhesion and extracellular matrix, morphogens and immunomodulatory factors^16^. Transcriptome analysis has identified differences in mRNA content between NG2-glia and oligodendrocytes, suggesting the existence of two individual glial cell populations^17^. This molecular diversity reflects the potential heterogeneity of the NG2-glia population based on either their location or on the developmental factors that could affect their activity^6^. In addition, there is significant heterogeneity among NG2-glia in terms of proliferation, differentiation and cell cycle rates^3,18,19^. However, it seems that the proliferative activity of NG2-glia is independent of their role in myelination, suggesting that this pool of cells may have other roles.

Like stem cells, NG2-glia can divide asymmetrically, preventing the exhaustion of NG2-cells in the adulthood^11,20^. In this respect, we previously reported significant clonal expansion of NG2-cells from SVZ pallial embryonic progenitors between P120 and P240^21^. Here, to specifically target the cell progeny derived from postnatal NG2-progenitors at the single cell level, we used the new StarTrack clonal analysis strategy, NG2-StarTrack^8^. Previously, NG2-EGFP-StarTrack was used to target the progeny of NG2-cells at several time points. Specifically targeting NG2-progenitors at embryonic and postnatal stages demonstrated their heterogeneous potential. Indeed, these progenitors can produce different neural cell types in the pallial cortex in vivo, depending on the embryonic or postnatal stage targeted. Here, we performed a clonal analysis to target individual postnatal progenitors (P0) with these piggyBac plasmids and we analyzed the cell progeny of these progenitors at different adult stages (P90, P240 and P365). Clonal analyses revealed that the labeled sibling cells produced larger clusters of NG2-cells in older animals, situated in the white matter (WM), and in both the grey matter (GM) and WM. Although a small percentage of clonally-related cells were identified as astrocytes, there was no clear clonal relationship between astrocytes and NG2-cells. Together, our results reveal new information regarding the fate potential of postnatal progenitors in relation to NG2-glia heterogeneity, as well as the clonal relationship of NG2-cell progeny in different pallial areas with age.

## Results

### StarTrack clonal analysis to decipher the progeny of postnatal NG2-progenitors

**Figure 1:**
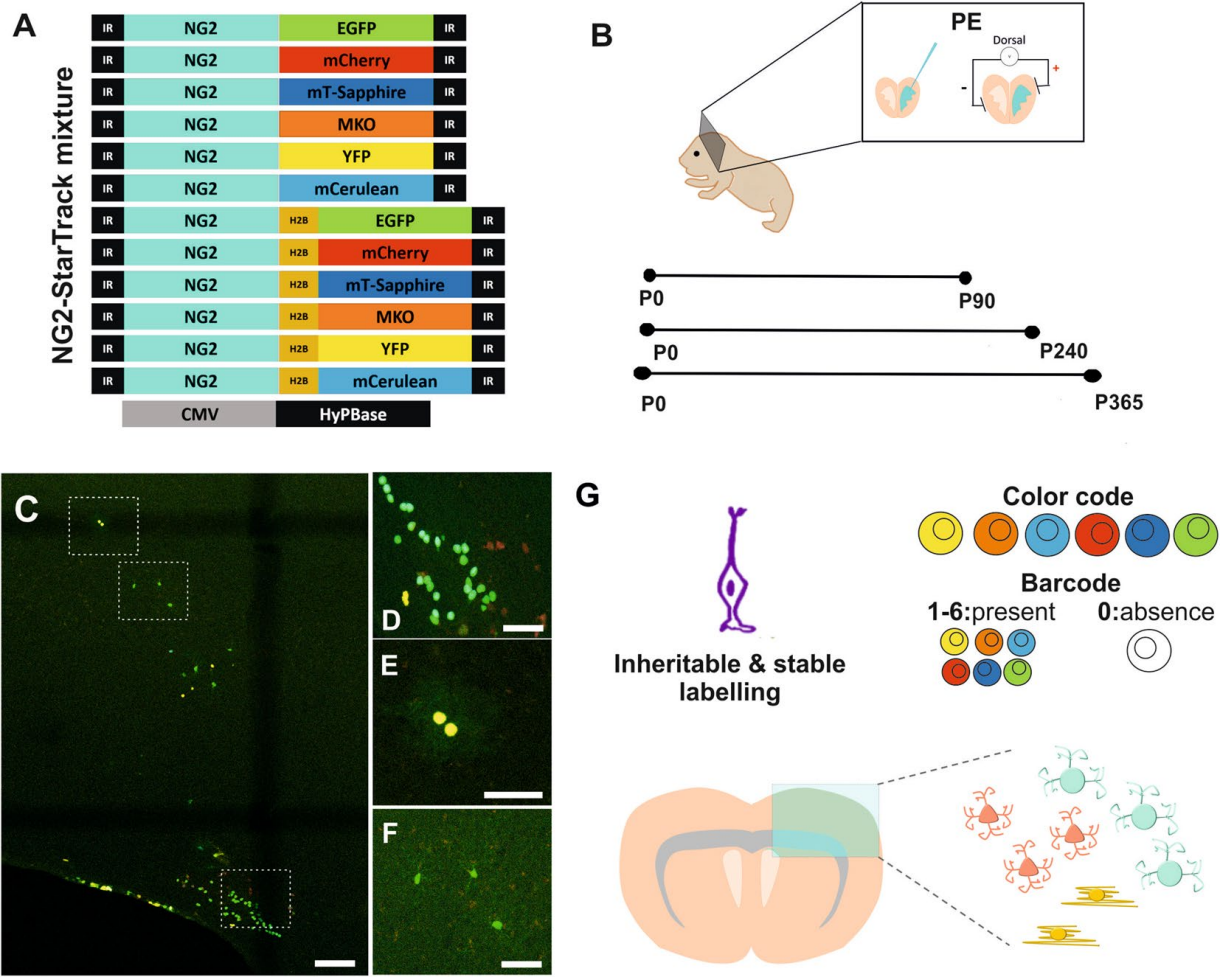
Clonal NG2-StarTrack approach. (**A**) Scheme of the twelve NG2-StarTrack piggyBac vectors along with the CMV-HyPBase transposase. The NG2-StarTrack contains inverted terminal repeats (IR) that the transposase recognizes, allowing it to randomly integrate copies of the NG2-StarTrack plasmids into the genome. (**B**) The consequences of postnatal electroporation at P0 were analyzed at different adult ages (P90, P240 and P365). (**C**) Targeted pallial pNSC produced different fluorescent cells in the cortex with immature morphologies, close to the LVs, as well as cells with different neural morphologies. White insets define the amplified images: (**D**) clonal related cells in the corpus callosum; (**E**) Small group of sibling cells in the GM; (**F**) Larger group of sibling cells in the GM. (**G**) Diagram of clonal analysis. Targeting single NSCs generates an inheritable and stable label in their progeny, creating a color and a barcode. The barcode is formed by a serial number (1–6) taking into account the presence or absence of XFPs and their location (cytoplasmic and nuclear). The labelled cells are widespread throughout the cerebral cortex along the rostro-caudal axis: *PE* postnatal electroporation, *CC* corpus callosum, *NSC* neural stem cell, *XFPs* different fluorescent proteins. Slice 50 µm. Scale bar 100 µm and 50 µm.

The StarTrack approach^22^ is based on 12 piggyBac plasmids that encode up to 6 different fluorophores (with either a nuclear or cytoplasmic localization), each driven by the GFAP-promoter, which are transfected along with a *piggyBac* transposase plasmid (hyPBase). This method allows single progenitor cells and their cell progeny to be targeted by piggyBac-driven stochastic integration into the genome. This strategy was modified to target the NG2 lineage in vivo by using novel plasmids carrying the mouse NG2-promoter (mNG2), referred to as NG2-StarTrack (Fig. 1A ^22^). Here, the mixture of NG2-StarTrack plasmids and the hyPBase transposase was injected into the lateral ventricles (LVs) of mice, which were electroporated at P0-P1 (Figs. 1B, S1). At P90, P240 and P365 we then performed a clonal analysis to assess the age-related changes in the postnatal NG2 derived cell progeny and in the progenitor cell potential.

NG2-StarTrack labelled cells in the dorsal cortex formed either big clusters, small groups or remained as individual cells (Fig. 1C). Different morphologies could be distinguished in WM (Fig. 1D) and GM (Fig. 1E,F), although the labeled immature cells located close to the ventricle were not considered in these clonal analyses. The cell progeny of targeted NG2-progenitors displayed an inheritable and stable color code at the single-cell level (Figs. 1G and S1). The different fluorescent reporter proteins were detected in separate channels to define the presence/absence of each fluorophore: 1, YFP; 2, mKO; 3, mCerulean; 4, mCherry; 5, mTSapphire; 6, EGFP (Fig. S1B). Each channel was assigned an emission color, except for mT-Sapphire that was represented as dark blue and far red in grey color. Accordingly, the cellular barcode allows the clonally-related cells to be rapidly recognized based on the presence (represented as 1–6) or absence (0) of the fluorescent proteins and their location, whereby the first number corresponds to cytoplasmic labeling and the second number to its nuclear location (Figs. 1G and S1C). Hence, the theoretical color-code created for each clonal cluster can produce more than 14,000 combinations^23^. Sequential sections along the rostro-caudal axis were used to analyze both the location and spatial dispersion of the sibling cells. The frequency of the different color-code combinations was also estimated to rule out the clones that appeared more frequently (data not shown).

### Characterization of the adult NG2 derived progeny of early postnatal progenitor cells

**Figure 2:**
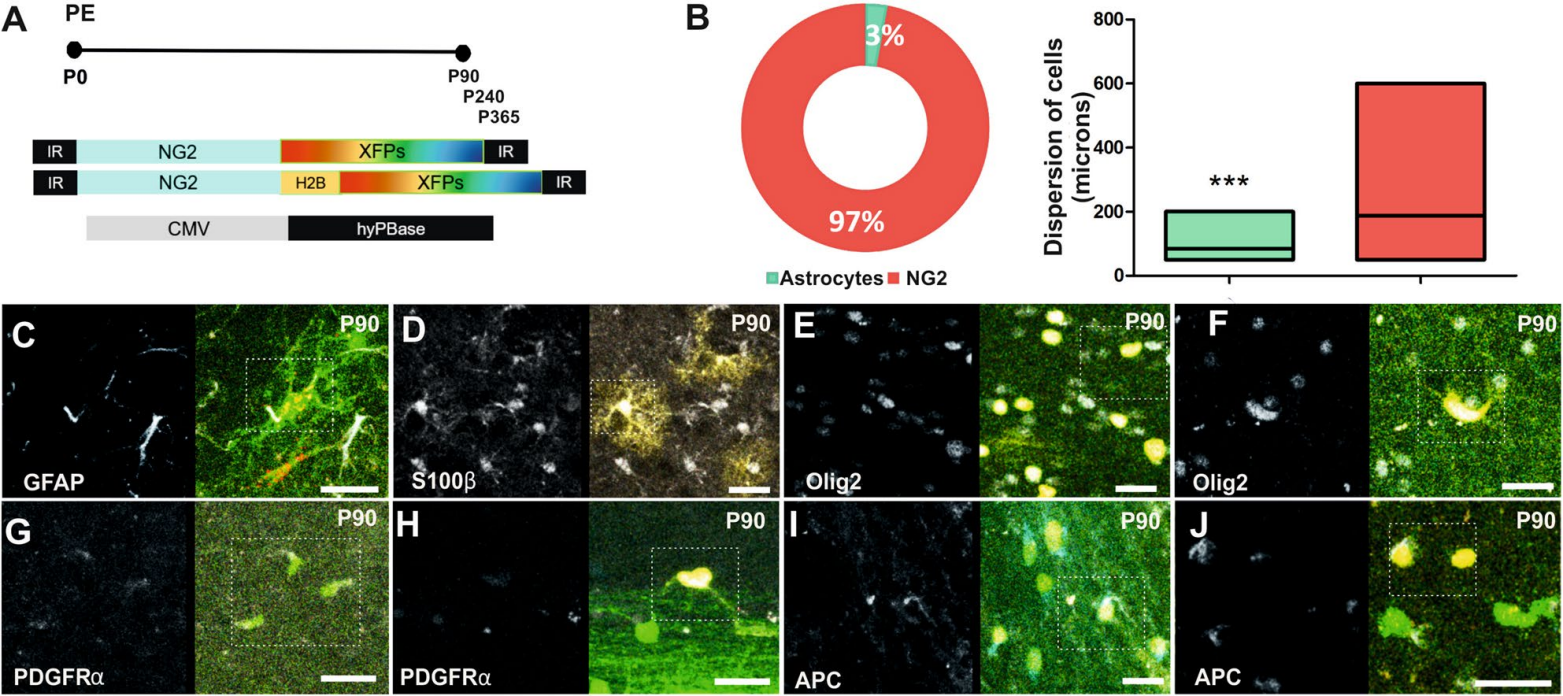
Cell derived progeny of targeted postnatal progenitor cells. (**A**) Scheme of the NG2-StarTrack approach. P0 pups were electroporated and analyzed at adult stages (P90; P240; P365). (**B**) NG2-cells represent 97% of the clonally related cells (4368 cells) and protoplasmic astrocytes just 3% (128 cells) in the total of 12 animals analyzed. The dispersion of total NG2-cells along the rostro-caudal axis was greater than that of astrocytes. Also, the dispersion of sibling NG2-cells relative to astrocytes showed significant differences. (**C**–**J**) Co-localization of neural markers with NG2-StarTrack labeled cells in the adult cerebral cortex (P90). (**C**, **D**) Astrocytes co-localize with GFAP and S100β. (**E**, **F**) Cluster of NG2-cells in the grey and white matter were identified with Olig2. (**G**, **H**) PDGFRα marked NG2- cells. (**I**–**F**) APC identified some immature oligodendrocytes: NG2-cells in red, astrocytes in green. Statistically significant differences are indicated by asterisks: *P < 0.05, **P < 0.01, ***P < 0.001. The line in the bars indicates the mean of data. Slices 50 µm. Scale bar: 20 µm.

NG2-StarTrack can target single progenitor cells with an active NG2 promoter and their derived cell progeny, identified using different neural markers at distinct adult ages (Fig. 2). The cell’s morphology and immunolabeling indicated the identity of the sibling cells, and among the 9 animals used in the clonal analysis (3 animals at each age) the proportion of NG2-cells was around 97% of the total cells analyzed (4496 of total cell analyzed) (Fig. 2B). This proportion was higher than that of the sibling astrocytes analyzed at all ages, which represented just 3% of the total cells (Fig. 2B). Similarly, the dispersion of these clonal astrocytes along the rostro-caudal axis was more restricted than that of the NG2 sibling clusters (Fig. 2B).

Although, the morphology of the labelled cells might be sufficient to identify the different types of glial cells, except for those with only nuclear labeling, we performed immunohistochemistry to assess the distribution of different glial markers. Labelled astrocytes were identified through the expression of both glial fibrillary acidic protein (GFAP: Fig. 2C) and S100 calcium binding protein beta (S100β: Fig. 2D). Cells in the NG2 lineage co-localized with labelling for Olig2 (Fig. 2E,F) and PDGFRα (Fig. 2G,H). The expression of NG2 is downregulated in oligodendrocytes, and some immature oligodendrocytes were labelled by these antibodies and identified by their expression of adenomatous polyposis Coli (APC/CC1: Fig. 2I–J). These cells did not express neuronal markers. After their characterization, the different groups of cells were classified in function of their cortical location, either in the GM, WM or both, revealing the identity of the progeny of dorsal postnatal progenitors after NG2-StarTrack targeting. The clonally-related cells in the GM and WM were mainly identified as NG2-cells, although some labelled astrocytes were detected in the GM. There were differences among the clusters of glial cells in terms of the number of cells and their dispersion along rostro-caudal axis.

### Clonal analysis of adult derived NG2-cell progeny after NG2-StarTrack targeting of individual postnatal progenitors

**Figure 3:**
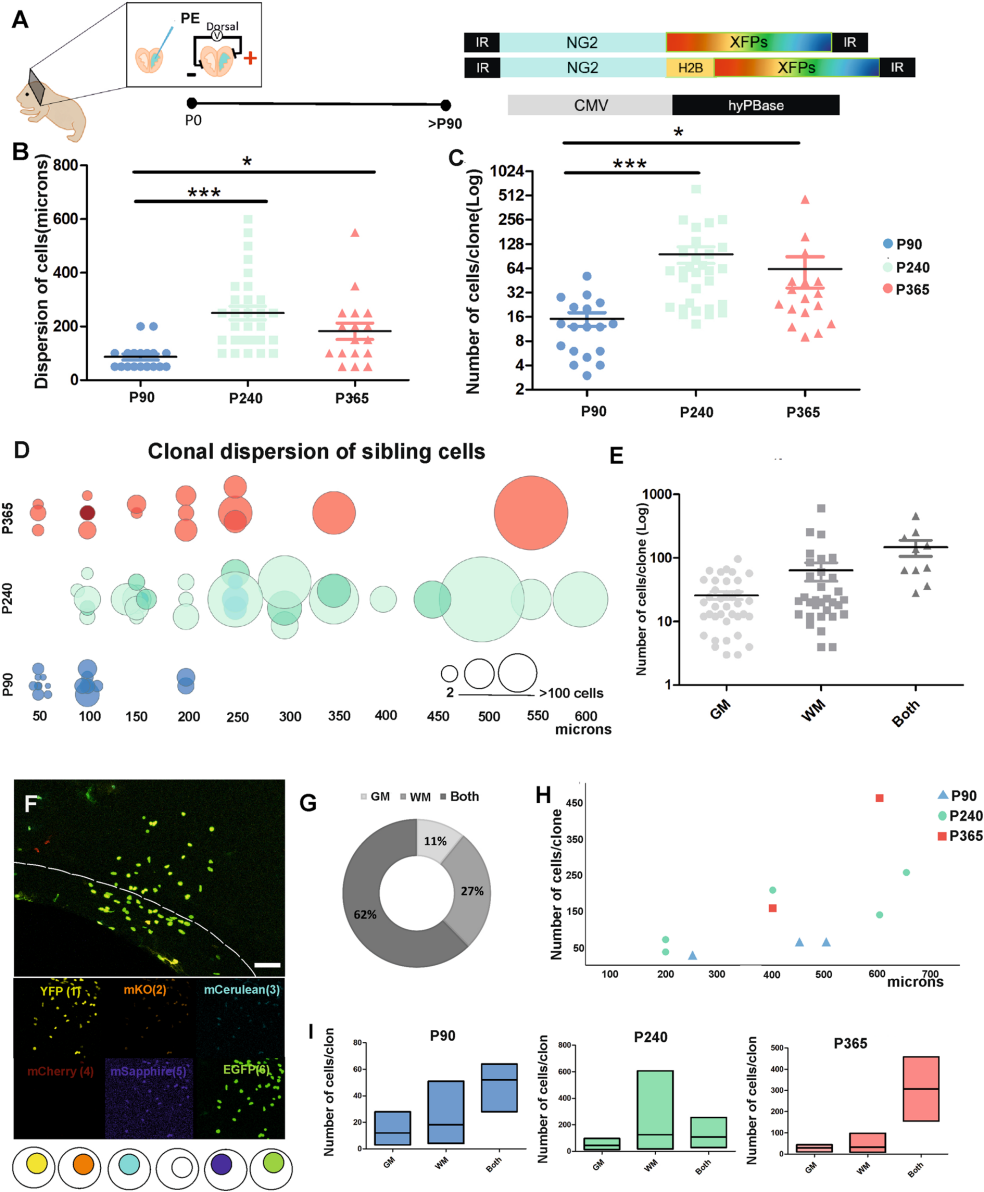
Clonal analysis and distribution of NG2-cells. (**A**) Diagram of the PE and NG2-StarTrack vectors. The tissue was analyzed from P90 onwards and clonal related cell progeny were located in dorsal areas of the brain. (**B**) The dispersion of the sibling NG2-cells increased with age. (**C**) The number of cells per clone was higher with age. (**D**) Clonal relationship of dispersion and the size of sibling cells at P90, P240 and P365. The dots show the different clonally related cells at each age and was normalized to the total number of cells per clone. (**F**) NG2-StarTrack labeled sibling cells in the corpus callosum and deeper layers of the cerebral cortex. The different channels show the presence/absence of the fluorescent protein and their locations (cytoplasmic or nuclear). Circles represent the color code of the clonal cells. (**G**) Proportion of sibling NG2-cells located in the GM, WM or both. (**H**) The dispersion of those clones was heterogeneous, from 150 to 650 μm depending on the clone size (fewer cells per clone was correlated with less dispersion along the rostro-caudal axis) A total of 10 clones were located in both areas (P90:3, P240:5 and P365:2). (**I**) Floating bars of clonal related NG2-cells in the GM, WM and both at the different time points. The larger clusters of sibling cells are located in both tissues at P90 and P365. The dark line in the bars and scatter dot-plot represents the mean. The data was represented as the mean ± SEM and the dots represented the different clones (**B**, **C**, **E**). P90 data is represented in blue, P240 in green and P365 in red. The GM is in soft grey, the WM in grey and Both are in dark grey. *PE* postnatal electroporation, *CC* corpus callosum, *GM* Grey matter, *WM* white matter; Both, grey and white matter; XFPs, different fluorescent proteins: *P < 0.05, **P < 0.01, ***P < 0.001. Slices 50 µm. Scale bar: 50 µm.

We then analyzed the clonal cell pattern of the pallial glial cells derived from postnatal (P0) progenitors with an active NG2 promoter (Fig. 3A) at different adult ages (P90, P240 and P365). We randomly selected 66 NG2-cell clones from three different animals of each age (P90:18, P240:30 and P365:18), attending to their color code and frequency. Their rostro-caudal location was calculated from the rostral end of the LV as the initial point of electroporation, and their dispersion was defined by the position of all the sibling cells at the different levels along the rostro-caudal axis. There was an increase in the number of cells per NG2 clone with age (Fig. 3C) and therefore, there was greater cell dispersion of most sibling NG2-cells (Fig. 3B), forming larger clones (Fig. 3C) with age (Fig. 3D). While the clones at P90 dispersed across 50–200 microns, in older animals the clones were larger and had a more heterogeneous dispersion. Hence, the biggest NG2 clone contained 607 cells at P240 (8 months) and the smallest just 2 cells (Fig. 3D). In addition, sibling NG2-cells were located in the GM, WM or both as mentioned above (Fig. 3E–I). At P240, the clones located in the WM and GM had more cells and a greater dispersion than at other ages (Fig. 3H,I). Nonetheless, the sibling cells located in the GM alone had fewer cells (Fig. 3E,G,I) and less cell dispersion than those in the WM. In addition, the presence of regionally mixed clones formed by NG2-cells located in both the GM and WM was notable, occupying the deeper cortical layers and the corpus callosum (Fig. 3F). Those clones located in both cortical areas constituted 62% of total labelled NG2-cells (Fig. 3G) displaying a huge cell dispersion, between 200 and 650 microns, at P240 and P365 (Fig. 3H). In this regard, the biggest clonal clusters were located in the WM (P240) or in both the WM and GM at the different ages analyzed (Fig. 3I). Together, the clonally-related NG2-cells formed larger clones in older animals, with an increase in cell dispersion. Moreover, some clonal cells were located in both the corpus callosum and the deeper layers of the dorsal cortex. These mixed clones were identified at all ages and they had more cells per clone.

### Clonal analysis of adult derived-astroglial progeny following NG2-StarTrack targeting of single postnatal progenitors

**Figure 4:**
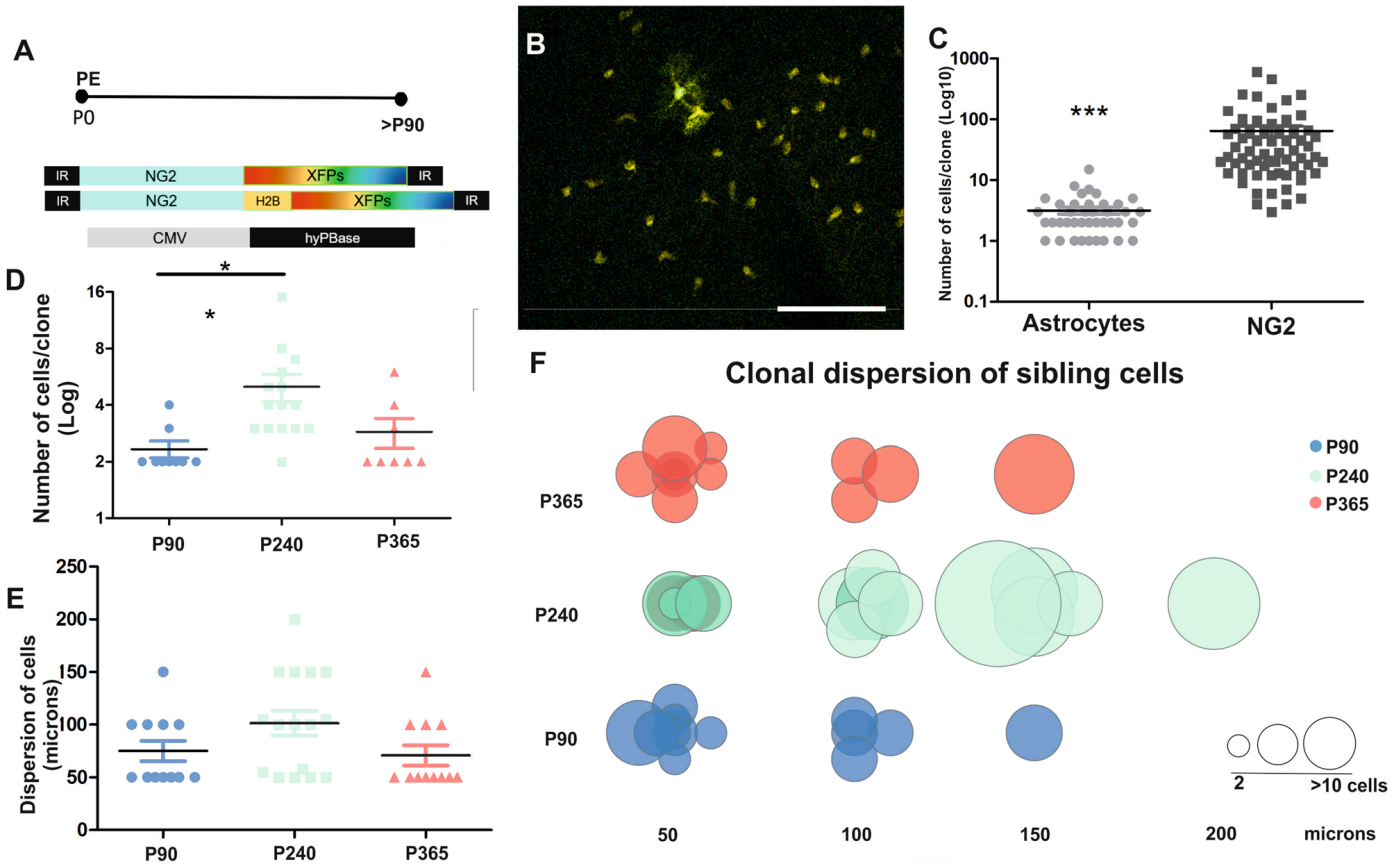
Clonal analysis and distribution of protoplasmic astrocytes. (**A**) Scheme of the postnatal electroporation and data analysis from P90 onwards. Diagram of the NG2-StarTrack mixture used to perform the clonal analysis. (**B**) Confocal imaging displayed two astrocytes surrounding by big group of sibling NG2-cells. (**C**) There were fewer of astrocytes than NG2-cells analyzed. (**D**) The number of clonal astrocytes increased at P240 but not at P365. (**E**) The dispersion of cells was constant with ageing. (**F**) Relationship of clonal dispersion and size of clonally-related astrocytes at P90, P240 and P365. The dots indicate the different clonal related cells per age and their size was normalized according to the number of sibling cells. The dispersion along the rostro-caudal axis was from 50 to 200 microns. The dark line in the bars and scatter dot-plot represents the mean, and the data was represented as mean ± SEM. The dots represent the different clones: P90 data is in blue, P240 in green and P365 in red. The dark line represents the mean in the dot plot graphs (**C**, **D**, **E**): *P < 0.05, **P < 0.01, ***P < 0.001. *PE* postnatal electroporation, *XFPs* different fluorescent proteins. Slices 50 µm. Scale bar: 50 µm.

**Figure 5:**
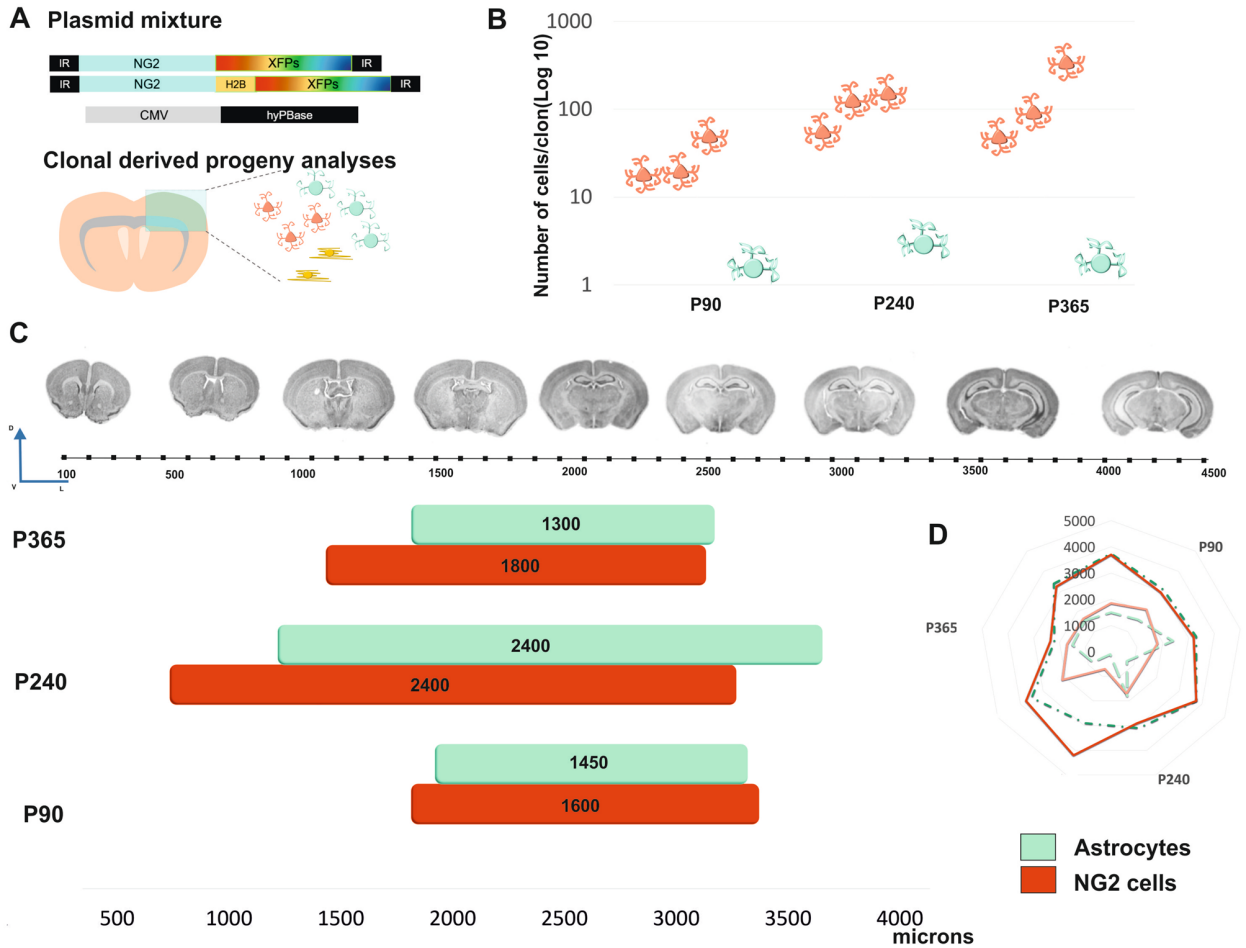
Summary of the temporal clonal analysis of NG2-derived cell progeny and their dispersion throughout the rostro-caudal axis. (**A**) Diagram of the NG2-StarTrack mixture and representation of the location of sibling cells in the adult brain. (**B**) Representative graph of the number of cells per clone with age, taking into account their cell identity. There are more NG2-cells with age whereas the number of astrocytes remains constant with age. (**C**) Dispersion of NG2 derived cell progeny along the rostro-caudal axis. Labeled cells were located between 500 to 4000 microns (starting from the beginning of the LVs). Astrocytes appeared caudally with respect NG2-cells at all ages but the dispersion pattern was similar (**D**). Astrocytes are in green and NG2-cells in red: *PE* postnatal electroporation, *XFPs* different fluorescent proteins, *LV* lateral ventricle.

After targeting pallial progenitor cells at P0, we designed a temporal clonal analysis (Fig. 4) to analyze the astrocytes labelled with the NG2-StarTrack at adult stages (P90, P240 and P365: Figs. 4A and 5A). From individual tagged postnatal NG2-progenitors we randomly selected 4368 adult labelled cells, from which 128 were identified as astrocytes in 40 clones located in the GM (P90:13; P240:15; P365:12). The number of fibrous astrocytes per clone was very low, usually just per one cell and thus, they were not considered in the clonal analysis. The cells were characterized based on both their morphology and marker expression (Figs. 4B and S1D,E). The astroglial clones displayed significant fewer cells per clone than the NG2 clones (Fig. 4B,C) and at P90, 8% of the labelled cells were astrocytes and 92% corresponded to NG2-glia. By contrast, astrocytes made up 2% of the tagged cells at P240 and P365. Comparing between ages, the number of protoplasmic astrocytes per clone at P90 was significantly different in older animals (Fig. 4D), with a maximum of 15 cells per clone at P240. Some labelled astrocytes with no sibling cells were observed at different ages (P90:3; P240:1; P365:3) and although yet they were not considered in the final clonal analysis, they could provide information about the behavior of early postnatal progenitors. In addition, the dispersion of astrocytes was apparently similar at all the ages selected (Fig. 4E,F). The majority of clonally-related astrocytes dispersed from 50 to 200 microns along the rostro-caudal axis (Fig. 4F), while the clonal size varied from 2 to 15 cells (Fig. 4D,F). Indeed, in relationship to the size of the clones, astrocytes exhibited homogeneous proliferation with age, in contrast to the NG2-glia clones in which the number of sibling cells increased in older animals (Fig. 5B). Comparing the cell distribution over the electroporated area (Fig. 5C), labelled astrocytes were preferentially located caudally to the NG2 labelled cells, which was most evident at P240 and P365 (Fig. 5C). Nevertheless, the distribution of these glial cells along the rostro-caudal axis was not significantly different (Fig. 5D). In summary, astroglial NG2-derived cell clones had a smaller dispersion and there were fewer cells per clone at the different ages analyzed, reflecting a homogeneous behavior of the tagged postnatal progenitor cells that contrasted with that of the progenitors that gave rise NG2-cells.

To conclude, we reveal that clones of NG2-cells in the cortical GM and WM show the largest cell dispersion at the different ages. In addition, we showed a lack of regional mixing of the clones of NG2-cells. These results witness the heterogeneity in both the postnatal NG2-progenitors and their cell progeny.

## Discussion

This study is a NG2-StarTrack clonal analysis of the adult cell progeny derived from individual NG2 pallial progenitors at early postnatal ages. To tag NG2- progenitor cells, we used the genomic multicolor genetic tracing tool, NG2-StarTrack, designed to track the cell progeny of individual postnatal progenitors from the SVZ in vivo^8^. Our data revealed the presence of clones of either NG2-glia or astrocytes with different spatio-temporal extensions. However, in contrast to embryonic NG2-progenitors^7,8,24^, no neuronal cells were derived from postnatal progenitors in cortical areas. Previous lineage analysis of neural cells reported the capability of progenitor cells to generate both astrocyte and oligodendrocyte cells in vitro^25–28^ and in vivo^29^.

NG2-cell clones were widespread throughout the cortical rostro-caudal axis, either in the WM and GM. In addition, a subpopulation of astroglial cells were tagged with the NG2-StarTrack mixture. These clones had a constant number of cells over time, unlike the NG2-cell clones that increased in number and dispersion. We also reveal the existence of regional mixed clones formed by clonally-related NG2-cells in both the cortical GM and WM. These mixed clones had more cells and a greater dispersion in the temporal analysis, arguing the NG2-cell heterogeneity may not only be related to their fate but also, to their location and to ontogenic processes^6,11^. Regional heterogeneity of NG2-cells has been reported, based on different properties like cell cycle length^30,31^, proliferative response^18^ and differentiation rates^32^. Even, NG2-cells in the same area present differences in terms of their transcription factor expression or that of the GPR17, G-protein couple receptor (GPCR^33^). In the adult brain, our lineage-tracing analyses of NG2-postnatal precursors revealed they proliferated distinctly and displayed different cell fate patterns across adulthood. As reported for the adult progeny of embryonic progenitors, the number of clonally-related cortical NG2-cells increases notably with age^21^. Nevertheless, some of those clones were in both the GM and WM, in contrast to embryonic progenitors. These postnatal progenitors proliferate and differentiate into NG2-cells, producing larger clones at older ages, and showing greater heterogeneity in terms of both cell dispersion and clonal size. This clonal expansion could be related to their proliferative activity over the animal’s lifetime and beyond the neurogenic niches^1,34,35^. Otherwise, other studies using Cre-lox mice reported that the proliferative rate of NG2-cells decreased with age^3–5^. As previously suggested using a different StarTrack approach^21^, it is possible that those pallial-derived NG2 clones increase their size at the expense of the direct differentiation in oligodendrocytes of ventral derived NG2 clones. Thus, those differences can be explained as a heterogeneous pool of progenitor cells with different proliferative properties throughout age and domain. Further, previous data reported that the mitotic status of adult NG2-cells is unrelated to their developmental origin^3^. However, there is a coexistence of both slow-cycling stem cell‐like NG2-cells with more rapidly cycling amplifying cells^3,20,30^, promoting that the cell cycle of NG2-cells varies in relation to the brain areas and age. Moreover, a smart approach on lineage-targeted transcriptomics reveals the role of transcription factors in lineage transition of glial cells in both physiologic and pathological conditions^36^. At this respect, complementary genetic approaches might assess new insights related to their NG2-cells heterogeneity and fate potential.

Our data show that postnatal progenitor cells gave rise to only glial lineages in cortical areas, while embryonic NG2-progenitors produce both neuronal and glial cell lineages^8^. In this regard, time-lapse imaging^37^ and in vivo StarTrack tracing^38^ revealed the impact of progenitor location on fate potential. Furthermore, it has been proposed that NG2-glia can differentiate into neurons under specific conditions and locations^39^. In this respect, several in vitro and in vivo approaches have been used to decipher the multipotent potential of progenitor cells and their capacity to generate different neural cell types^40,41^. Cre-inducible mouse lines produced few astrocytes after induction^19^, but in other transgenic mice, no astrocytes or neurons were found at adult stages^42^. All these differences suggest the need to focus on progenitor heterogeneity^8,43,44^ which is essential to address the cell fate of neural cells.

NG2-glia generate astrocytes during development^7,44,45^, although it remains unclear if that capacity persists in adults^39,46,47^. The small subpopulation of astroglial cells in relation the number of NG2-cells, derived from NG2 postnatal progenitors, revealed that these cells have different proliferation rates related not only to age but also, to their origin. Clonal astrocytes were less abundant than NG2-cells at all stages and their cell dispersion in the rostro-caudal axis was homogeneous. Thus, as well NG2-glia, astrocytes are characterized by their heterogeneity at different levels, including ontogeny^22,48^, morphology^49,50^, cortical GM and WM location^22,51^, transcriptomic signatures^52,53^ or response to brain lesions^54,55^. In addition, astrocytes could influence NG2-glia behavior during development, an interaction that may have an important influence on gliogenesis, ageing and even injury^56^. Recently, sibling astrocytes were shown to preferentially establish GAP-junction coupling relative to the unrelated astrocytes^57^. This coupled response between sibling cells may be implicated in physiological and pathological events both, astrocytes and NG2-glia^55,58^. All these data reinforce the strong heterogeneity of these neural cells at a morphological, functional and genetic level, opening the window to develop new lineage tracing approaches not only based on multicolor labeling but also, on stable genome editing^59^. New transcriptome tools could shed light on the pathologies mechanisms and further therapies^60^.

Ageing is related to the loss of myelin, which is in turn correlated with the loss of cognitive and motor skills, which could be a consequence of the generation of fewer oligodendrocytes^61^. However, NG2-cells preserve the ability to divide in adulthood^62,63^ and moreover, NG2-glia respond early to brain insults (like astrocytes), migrating towards to the injury site and increasing their rate of proliferation^19,25^. Several reports indicate that most NG2-cells in the cortical GM could be part of a different population of NG2-cells under physiological conditions^24^, although their functions are not fully understood. Thus, NG2-cells increase in number with age, which may be useful to design new therapies to combat cell aging related phenomena. Their capability to generate neurons is not yet fully understood, yet their stem cell like characteristics or their possible reprogramming could be relevant to neurodegenerative diseases and ageing^12,13,64^.

In conclusion, beyond their role in myelination, NG2-glia represent a significant pool of glial cells with diverse functionalities, as well as unique properties that contribute to CNS homeostasis and development. The importance of their origin, cell fate and heterogeneity is still unclear. Thus, further clonal analysis complemented with genetic cell identity strategies might help gain new insights into their behavior, heterogeneity and fate potential.

## Materials and methods

### Animals

All mice were maintained under standard housing conditions at the animal facility of the Cajal Institute. All procedures involving animals were carried out in accordance with the European Union guidelines on the use and welfare of experimental animals (2010/63/EU) and those of the Spanish Ministry of Agriculture (RD 1201/2005 and L 32/2007). All the experiments were approved by the CSIC Bioethical Committee (PROEX 223/16). Both male and female of C57/BL6 mice were used indistinctly as we have not observed any difference associated with sex in the biological processes studied. The day of birth was considered as postnatal day 0 (P0) and in all the experiments, a minimum of n = 3 animals was studied for each condition.

### Vectors

NG2-StarTrack constructs were designed as described previously^8^. Briefly, the GFAP-StarTrack constructs were used to generate the different NG2 piggyBac vectors with the six different reporter proteins and H2B histone sequence to drive the tag into the nucleus. The human GFAP promoter was removed and replaced with a murine NG2 promoter^65^. The hyperactive transposase of the PiggyBac system (CMV-hyPBase) was kindly provided by Dr Bradley and all the plasmids used were sequenced to confirm successful cloning (Sigma–Aldrich; Merck KGaA, Darmstadt, Germany). For all injections, the plasmid mixtures contained the twelve NG2-StarTrack constructs and a hyperactive transposase of the PiggyBac system under the CMV promoter to integrate copies of the NG2-StarTrack plasmids randomly into postnatal progenitor cells.

### Postnatal electroporation

Postnatal electroporation was performed as described previously^38^. Briefly, a plasmid solution containing the plasmid mixture (1–2 µg/µl) and 0.1% Fast Green was injected into the LVs of perinatal animals on day P0-1 using a glass micropipette. After plasmid injection, all pups were electroporated with electrode paddles, placing electroconductive LEM Gel (DRV1800, MORETTI S.P.A.) on both paddles to avoid damage to the pups and to achieve successful current flow. We administered five pulses of 100 V, each pulse lasting 50 ms and separated by 950 ms intervals. The positive electrode was positioned on top of the dorsal cortex to direct the negatively charged DNA. After the five pulses, the electroporated animals were reanimated for several minutes on a 37 °C heating plate before returning them to the mother’s nest. The mice were analyzed from P30 onwards (at least three animals per experimental group).

### Histological and immunohistological procedures

The adult animals were anesthetized with pentohbarbital (Dolethal, 40–50 mg/Kg) and when fully anesthetized, they were perfused with 4% paraformaldehyde (PFA), and their brain was removed and placed overnight in small tubes with 4% PFA in 0.1 M phosphate buffer (PB). Serial vibratome brain section (50 µm thick) were mounted onto glass slides with Mowiol and stained for different neural markers. Slices were permeabilized with phosphate buffer saline containing Triton X-100 (PBS-T) and then incubated for at least 30 min in blocking solution (5% normal goat serum-NGS- in PBS-T 0.1%). The sections were then incubated O/N at 4 °C with the following antibodies markers: rabbit polyclonal anti-Olig2 (Millipore-AB9610); rabbit polyclonal anti-GFAP (Dako-31745); mouse monoclonal anti-APC (Calbiochem (OP80); rabbit polyclonal anti-PDGFRα (Cell Signalling-3169); a mouse monoclonal anti-S100β (Abcam-Ab66028). After at least three washes with buffer, the sections were incubated for up to 2 h with Alexa far red goat anti-rabbit or goat anti-mouse IgG (1:1.000, Alexa Fluor 633 or 647, Molecular Probes). Finally, the sections were washed several times with buffer, mounted on slides, coverslipped and observed in an epifluorescence microscope (Eclipse E600; Nikon, USA).

### Imaging acquisition

The sections were examined under an epifluorescence microscope equipped with GFP (FF01-473/10), mCherry (FF01-590/20) and Cy5 (FF01-628/40-25) filters. Images were then acquired on a TCS-SP5 confocal microscope (Leica, TCS-SP5). The confocal laser lines were maximal around 40% in all samples and the conditions for each laser was constant for each animal. The different reporter proteins were taken in separate channels controlling the overlapping between them. The wavelength of excitation (Ex) and emission (Em) was (in nanometers): mT-Sapphire (Ex: 405; Em: 520–535), mCerulean (Ex: 458; Em:468–480), EGFP (Ex:488; Em: 498–510), YFP (Ex:514; Em: 525–535), mKO (Ex: 514; Em: 560–580), mCherry (Ex: 561; Em: 601–620), and Alexa Fluor 633/647 (Ex: 633; Em: 650–760). Maximum projection images were analyzed using LASX software (Leica) and Fiji software ImageJ. All stitching and contrast adjustments were performed with the LasX software (LasX Industries) and Photoshop CS5 software (Adobe). The rostro-caudal axis was reconstructed using the Traken2 plug-in for ImageJ and was estimated as the distance between the beginning of the LVs and the last slice containing cells of that clone. Moreover, the clonal dispersion was calculated considering the distance between the first and last slice containing clonally related cells.

### Data analysis

For each experiment, the sections were analyzed serially and the cells were counted using the manual cell counter plug-in of ImageJ software. Afterwards, the proportion of those cells in the pallial areas was calculated. For statistics, GraphPad Prism 6.0 (GraphPad, USA) was used and the statistical significance between two groups was assessed with two-tailed unpaired Student’s *t*-tests, using ANOVA for multiple comparisons between the groups. The values were represented as mean ± SEM along the experimental data. A confidence interval of 95% (*p* < 0.05) was determined for the statistically significant values. Critical values of *p < 0.05, **p < 0.01, and ***p < 0.001 were adopted to determine statistical differences. Graphs were obtained using Excel Office, GraphPad Prism 6.0 (San Diego, USA) and CorelDRAW Graphic Suite 2018 (Corel Corporation, Ottawa, Canada).

Clonal analysis was performed on numbered cells and examined through the presence/absence of fluorophore and their location. A barcode was created as a binary signature (0 = absence, 1 = presence of cytoplasmic and nuclear marker of YFP, mKO, mCerulean mCherry, mTSapphire and EGFP) in all the animals analyzed. Since cell labeling and intensity in the maximal projection depend on the z-position of the cells, that could not be constant in relation to the intensity of the different reporter proteins, we followed specific criteria to avoid misleading. When the labelled cell does not exhibit a clear label of the cell nucleus, we just considered that this corresponds to a cytoplasmic labeling. On the other hand, when there is a clear limit discriminating the nucleus and the cytoplasm, within the same fluorescent channel, we consider the labeling to be both nuclear and cytoplasmic. Finally, the cells sharing the same combination/location of fluorophores/signature were catalogued as clones after classifying all the labeled cells.

## Supplementary information

Supplementary Information.
