# Supplementary material for: Unraveling the adult cell progeny of early postnatal progenitor cells
Source: Sci Rep. 2020 Nov 4;10:19058. doi: 10.1038/s41598-020-75973-y (PMC7643156; doi:10.1038/s41598-020-75973-y)
Supplement: Supplementary file 1 — Supplementary Information. [file 41598_2020_75973_MOESM1_ESM.pdf]

Supplementary Information

Manuscript Type: Original Research

Unraveling the Adult Cell Progeny of Early Postnatal Progenitor Cells

Rebeca Sánchez-González, Nieves Salvador and Laura López-Mascaraque

Instituto Cajal-CSIC, 28002 Madrid, Spain.

Figure S1

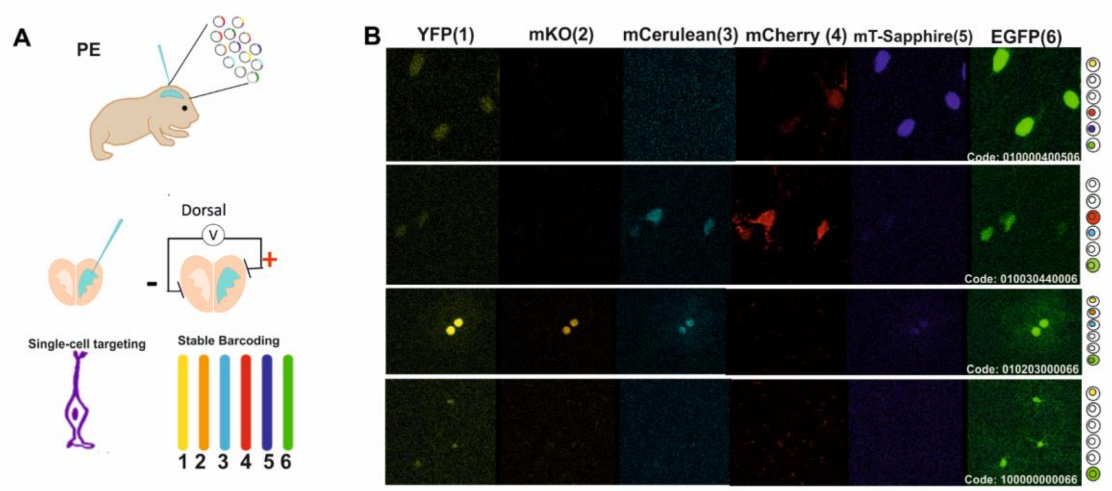

C

Clon	YFP	Mko	mCerulean	mCherry	mTSapphire	EGFP	Code	Microns	Nº of cells	Location	Matter	Glial Cell
1	1	2	0	0	0	0	010200000000	150	7	Dorsal cortex	G	Astrocyte
2	1	0	30	0	0	6	010030000006	100	5	Dorsal cortex	G	Astrocyte
3	11	22	0	0	0	0	112200000000	150	6	Dorsal cortex	G	Astrocyte
4	10	20	30	0	0	0	010203000000	105	5	Dorsal cortex	G	Astrocyte
5	1	20	0	40	0	0	012000400000	105	3	Dorsal cortex	G	Astrocyte
6	11	22	33	44	5	66	112233440566	50	1	Dorsal cortex	G	Astrocyte
7	10	0	0	0	0	6	100000000006	58	3	Dorsal cortex	G	Astrocyte
8	10	20	0	0	0	0	102000000000	150	15	Dorsal cortex	G	Astrocyte
9	10	20	0	0	5	60	102000000560	100	4	Dorsal cortex	G	NG2cells
10	0	0	3	0	0	0	000030000000	100	13	Dorsal cortex	W	NG2cells
11	0	0	3	0	0	6	000003000006	100	12	Dorsal cortex	W	NG2cells
12	1	2	0	4	0	6	010200040006	50	4	Dorsal cortex	G	NG2cells
13	1	2	3	0	0	0	010203000000	50	4	Dorsal cortex	W	NG2cells
14	0	0	3	0	0	0	000030000000	200	28	Dorsal cortex	W+G	NG2cells
15	1	2	0	0	0	0	100200000000	50	12	Dorsal cortex	G	NG2cells
16	0	0	0	4	0	6	000000400006	550	458	Dorsal cortex	W+G	NG2cells
17	1	0	3	0	0	6	010030000006	350	156	Dorsal cortex	W+G	NG2cells
18	0	0	3	0	0	0	000000300000	200	43	Dorsal cortex	W	NG2cells
19	1	2	0	4	0	6	010200040006	50	4	Dorsal cortex	G	NG2cells

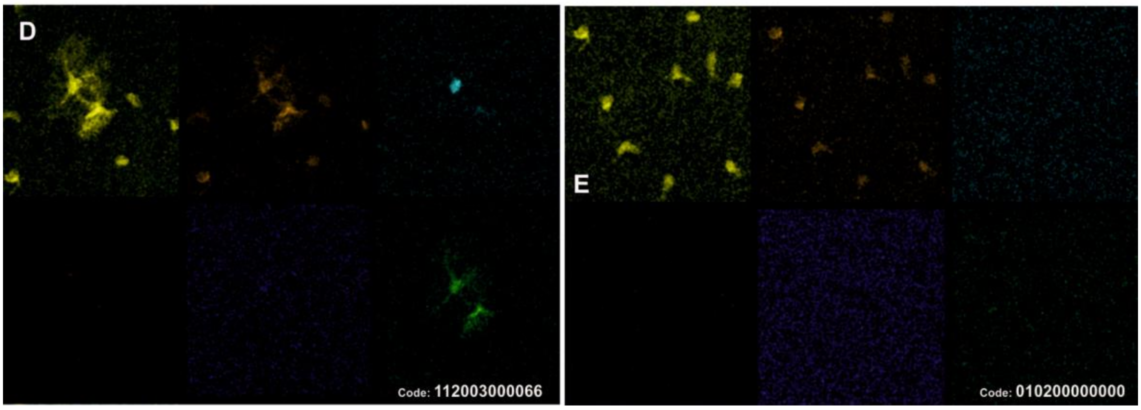

Figure S1. Clonal analysis of adult NG2 cell progeny. (A) Diagram of the PE procedure with the NG2-StarTrack mixture to target single-progenitor cells at P0 and create a stable and inheritable labeling that allow us to follow their cell progeny. **(B)**Separate channels of the different fluorescent proteins enable us to reconstruct the color codes of each cell and determine the final barcode depending on their location (cytoplasmic/nuclear) and the presence/absence of the reporter protein. **(C)** Table of quantitative analysis of number of cells per clon according with their barcode and dispersion of clonal related cells. Astrocytes are in soft green and NG2 cells in red. **(D)** Clonal related astrocytes channel capture showed the color code and the resulting barcode take into account the presence/absence of fluorophore and their location in the cell. **(E)** Clonal related NG2-glia in separate channels displayed a common color and barcode for all sibling cells. PE: Postnatal electroporation. Slice: 50 μm
